# An Effective Multimodal Image Fusion Method Using MRI and PET for Alzheimer's Disease Diagnosis

**DOI:** 10.3389/fdgth.2021.637386

**Published:** 2021-02-26

**Authors:** Juan Song, Jian Zheng, Ping Li, Xiaoyuan Lu, Guangming Zhu, Peiyi Shen

**Affiliations:** ^1^School of Computer Science and Technology, Xidian University, Shaanxi, China; ^2^Data and Virtual Research Room, Shanghai Broadband Network Center, Shanghai, China

**Keywords:** Alzheimer's disease, multimodal image fusion, MRI, FDG-PET, convolutional neural networks, multi-class classification

## Abstract

Alzheimer's disease (AD) is an irreversible brain disease that severely damages human thinking and memory. Early diagnosis plays an important part in the prevention and treatment of AD. Neuroimaging-based computer-aided diagnosis (CAD) has shown that deep learning methods using multimodal images are beneficial to guide AD detection. In recent years, many methods based on multimodal feature learning have been proposed to extract and fuse latent representation information from different neuroimaging modalities including magnetic resonance imaging (MRI) and 18-fluorodeoxyglucose positron emission tomography (FDG-PET). However, these methods lack the interpretability required to clearly explain the specific meaning of the extracted information. To make the multimodal fusion process more persuasive, we propose an image fusion method to aid AD diagnosis. Specifically, we fuse the gray matter (GM) tissue area of brain MRI and FDG-PET images by registration and mask coding to obtain a new fused modality called “GM-PET.” The resulting single composite image emphasizes the GM area that is critical for AD diagnosis, while retaining both the contour and metabolic characteristics of the subject's brain tissue. In addition, we use the three-dimensional simple convolutional neural network (3D Simple CNN) and 3D Multi-Scale CNN to evaluate the effectiveness of our image fusion method in binary classification and multi-classification tasks. Experiments on the Alzheimer's Disease Neuroimaging Initiative (ADNI) dataset indicate that the proposed image fusion method achieves better overall performance than unimodal and feature fusion methods, and that it outperforms state-of-the-art methods for AD diagnosis.

## 1. Introduction

Alzheimer's disease (AD) is a progressive brain disorder and the most common cause of dementia in later life. It causes cognitive deterioration, eventually resulting in inability to carry out activities of daily life. AD not only severely degrades patients' quality of life but also causes additional distress for caregivers (1). At least 50 million people worldwide are likely to suffer from AD or other dementias. Total payments in 2020 for health care, long-term care, and hospice services for people aged 65 and older with dementia are estimated to be $305 billion (2). And the number of AD patients is estimated to be 115 million by 2050. Therefore, accurate early diagnosis and treatment of AD is of great importance.

Currently, the pathogenesis of AD is not fully understood. The academic community generally believes that AD is related to neurofibrillary tangles and extracellular amyloid-β (Aβ) deposition, which cause loss or damage of neurons and synapses (3, 4). In general, the AD diagnostic system classifies a subject into one of three categories: AD, mild cognitive impairment (MCI), and normal control (NC). The main clinical examination methods for AD include neuropsychological examination and neuroimaging examination (5), in which computer-aided diagnosis is of great help in screening at-risk individuals. Psychological auxiliary diagnosis of AD uses the Mini-Mental State Examination (MMSE) and Clinical Dementia Rating (CDR) to help clinicians determine the severity of dementia. With the rapid development of neuroimaging technology, neuroimaging diagnosis has become an indispensable diagnostic method for AD. In particular, magnetic resonance imaging (MRI) and positron emission tomography (PET) are popular and non-invasive techniques used to capture brain tissue characteristics.

Structural MRI has become a commonly used structural neuroimaging in AD diagnosis because of its high resolution for soft tissue and its ability to present brain anatomical details. Progression of AD results in gross atrophy of the affected regions, including degeneration in the temporal lobe and parietal lobe, as well as parts of the frontal cortex and cingulate gyrus (6). Brain ventricles, which produce cerebrospinal fluid (CSF), become larger in AD patients. And the brain cortex shrivels up, with severe shrinkage occurring particularly in the hippocampus area. MRI, which provides three-dimensional (3D) images of brain tissues, enables clear observation of these structural changes in the patient's brain. Notable results were reported by a number of studies of clinical diagnosis of AD using MRI. Klöppel et al. (7) first segmented the whole brain into gray matter (GM), white matter (WM), and CSF, and used GM voxels as features of MR images to train a support vector machine to discriminate between AD and NC subjects. Owing to the strong relationship of GM with AD diagnosis, compared with WM and CSF (8, 9) only considered spatially normalized GM volumes, called GM tissue densities, for classification. Similarly, Zhu et al. (10) only computed the volume of GM as a feature for each region of the 93 regions of interest in the labeled MR image and used multiple-kernel learning to classify the neuroimaging data. These studies indicate that GM tissue is the most important area for AD classification using MRI (11, 12).

PET imaging has a critical role as a functional technique that enables clinicians to observe activities related to the human brain quickly and precisely, with particular applications in early AD detection (13). As stated in (14), PET images captured via diffusion of radioactive 18-fluorodeoxyglucose (FDG) have been used to obtain sensitive measurements of cerebral metabolic rates of glucose (CMRglc). CMRglc can be used to distinguish AD from other dementias, predict and track decline from NC to AD, and screen at-risk individuals prior to the onset of cognitive symptoms. FDG-PET is particularly useful when changes in physiological and pathological anatomy are difficult to distinguish (15). For instance, the volume of brain structures commonly decreases with age (e.g., in individuals older than 75 years), making it difficult to determine whether a person's brain is in a normal or diseased state only using the brain anatomical changes observed by MRI. In such cases, PET can more effectively detect the disease status of subjects.

Structural MRI can reflect the changes of brain structure, whereas functional PET images can capture the characteristics of brain metabolism to enhance the ability to find lesions (16). Therefore, it has been proposed that multimodal methods combining MRI and PET images could improve the accuracy of AD classification (17–19). Feature fusion strategies are commonly used in multimodal learning tasks, combining high-dimensional semantic features extracted from different unimodal data (20, 21). For example, Shi et al. (22) used two stacked deep polynomial networks (SDPNs) to learn high-level features of MRI and PET images, respectively, which were then fed to another SDPN to fuse the multimodal neuroimaging information. Similarly, Lu et al. (23) used six independent deep neural networks (DNN) to extract corresponding features from different scales of unimodal images (such as those obtained by MRI or PET); the features were then fused by another DNN. Related studies show that a feature fusion strategy can indeed achieve better experimental performance than use of unimodal data alone (24, 25). However, such a method is a “black box,” lacking sufficient interpretability to explain the exact reason for better or worse results in a particular case. In addition, deep learning methods based on feature fusion always greatly increase the number of model parameters, as a multi-channel input network is used to extract heterogeneous features from different modalities.

Compared with feature fusion strategies, multimodal medical image fusion is a more intuitive approach that integrates relevant and complementary information from multiple input images into a single fused image in order to facilitate more precise diagnosis and better treatment (26). The fused images have not only richer modal characteristics but also more powerful information representation. Besides, GM is the most important tissue for AD auxiliary diagnosis, which can show the brain's anatomical changes in MRI scans and the overall level of brain metabolism in PET scans. Motivated by these factors, we propose an image fusion method that fuses GM tissue information from MRI and FDG-PET images into a new GM-PET modality. During the fusion process, only the key GM areas are preserved, instead of the full MRI and PET information, to reduce noise and irrelevant information in the fused image and enable the subsequent feature extraction to focus on the crucial characteristics.

The main contributions of this work are two-fold. (1) A novel image fusion method is proposed for AD diagnosis to enhance the information representation ability of neuroimaging modalities by fusing the key GM information from MRI and PET scans into a single composite image. (2) We propose two 3D CNN for AD diagnosis, i.e., 3D Simple CNN and 3D Multi-Scale CNN, to evaluate the performance of different modalities in AD classification tasks. We also prove that the proposed fused modality with its powerful information representation can provide better diagnostic performance and adapt to different CNN.

The rest of this paper is organized as follows. section 2 describes the dataset used and our image fusion method. Our 3D Simple CNN and 3D Multi-Scale CNN are introduced in section 2.3 to extract the features and perform classification based on the neuroimaging data. In section 3, classification experiments for AD vs. NC, MCI vs. NC, AD vs. MCI, and AD vs. MCI vs. NC are conducted to evaluate the effectiveness of our proposed image fusion in an AD diagnostic framework. The discussion and conclusion are presented in sections 4 and 5, respectively.

## 2. Materials and Methods

### 2.1. Datasets

The data used in the study were acquired from the Alzheimer's Disease Neuroimaging Initiative (ADNI) dataset (https://adni.loni.usc.edu/). ADNI is a longitudinal multicenter study designed to develop clinical, imaging, genetic, and biochemical biomarkers for the early detection and tracking of AD. ADNI makes all data and samples available for scientists worldwide to promote AD diagnosis and treatment (27, 28). The ADNI researchers have collected and integrated analyses of multimodal data, mainly from North American participants. The dataset contains data from different AD stages. In this study, subjects were selected who had both T1-weighted MRI and FDG-PET scans captured in the same period. MRI scans labeled as MPRAGE were selected as these are considered the best with respect to quality ratings. A total of 381 subjects from the ADNI were selected, comprising 95 AD subjects, 160 MCI subjects, and 126 NC subjects. Clinical information for the selected subjects is shown in Table 1.

**Table 1:** Demographic information for subjects. Values are presented as mean ± standard deviation.

**Subjects**	**Number**	**Male/ Female**	**Age**	**MMSE**	**CDR**
NC	126	71/55	75.25 ± 5.82	29.58 ± 0.66	0.02 ± 0.18
MCI	160	108/52	76.97 ± 8.23	26.14 ± 0.81	1.38 ± 2.00
AD	95	54/41	76.52 ± 6.96	18.56 ± 4.20	2.87 ± 3.60

The MRI and FDG-PET images in ADNI have undergone several processing steps. In detail, the MRI images are processed by the following steps: Gradwarp, B1 non-uniformity, and N3. Gradwarp corrects image geometry distortion caused by the gradient model, and B1 non-uniformity corrects image intensity non-uniformity using B1 calibration scans. Finally, an N3 histogram peak-sharpening algorithm is applied to reduce the non-uniformity of intensity. The need to perform the image pre-processing corrections outlined above varies among manufacturers and system RF coil configurations. We used the fully pre-processed data in our experiments.

In order to obtain more uniform PET data among different systems, the baseline FDG-PET scans are processed by the following steps. (1) Co-Registered dynamic: six 5-min FDG-PET frames are acquired within 30–60 min post-injection, each of which is co-registered to the first extracted frame. The independent frames are co-registered to one another to lessen the effects of patient motion. (2) Averaging: six co-registered frames obtained are averaged. (3) Standardization of image and voxel size: the averaged image is reoriented into a standard 160 × 160 × 96 voxel image grid with 1.5 mm cubic voxels after anterior commissure–posterior commissure correction, followed by intensity normalization using a subject-specific mask so that the average value of voxels within the mask is exactly one. (4) Uniform resolution: the normalized image is filtered with a scanner-specific filter to obtain an image with a uniform isotropic resolution of 8 mm full width at half maximum, in order to smooth the above-mentioned images.

### 2.2. Proposed Image Fusion

**Figure 1:**
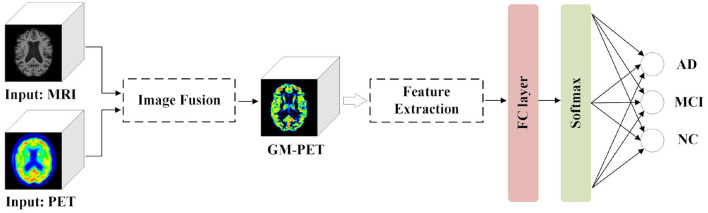
Proposed AD diagnostic framework with multimodal image fusion method.

To make the multimodal fusion process more interpretable, we propose fusing MRI and PET scans at the image field. The fused image modality is then fed into a single-channel network for diagnosis of subjects. This approach greatly reduces the number of model parameters compared with the multi-channel input network using feature fusion. Our proposed AD diagnostic framework with multimodal image fusion method is presented in Figure 1. It is composed of several parts: image fusion, feature extraction, and classification. First, our image fusion method can obtain a new GM-PET modality from the MRI and PET images. Subsequently, the semantic features are extracted from the GM-PET images. Finally, the classifier consisting of a fully connected (FC) layer and a softmax layer is used to classify subjects from different groups.

**Figure 2:**
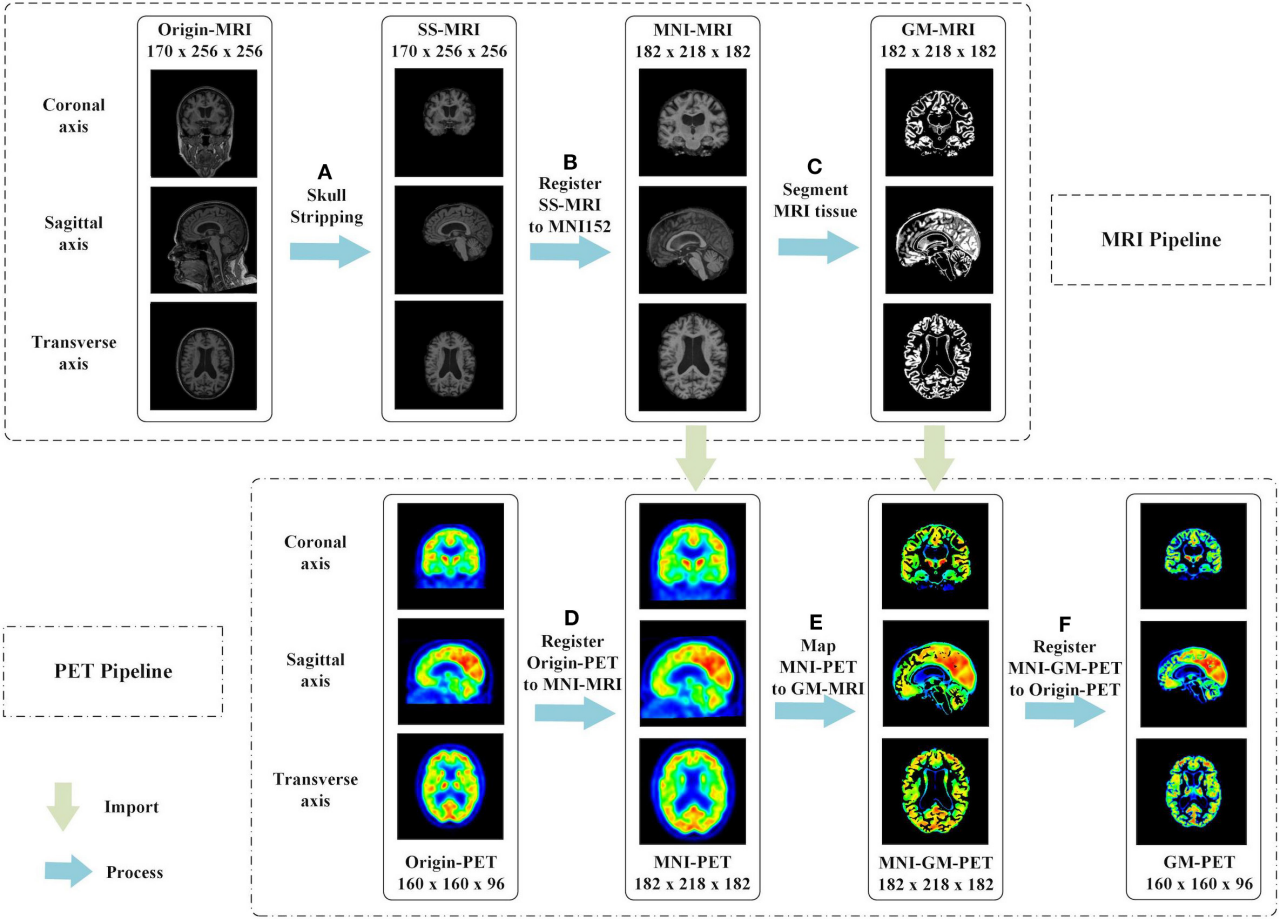
Proposed multimodal image fusion method. In the MRI pipeline, we executed the following steps in sequence: **(A)** skull-stripping, **(B)** registration of SS-MRI to MNI152, and **(C)** segmentation of MRI tissue. The phased output of the MRI pipeline guided the subsequent processing of PET images, as shown by the green arrows. In the PET pipeline, we performed the following steps: **(D)** registration of Origin-PET to MNI-MRI, **(E)** mapping MNI-PET to GM-MRI, and **(F)** registration of MNI-GM-PET to Origin-PET.

The proposed multimodal image fusion can merge complementary information from different modality images so that the composite modality conveys a better description of the information than the individual input images. As depicted in Figure 2, our proposed image fusion method only extracts the GM area that is critical for AD diagnosis from FDG-PET, using the MRI scan as an anatomical mask. The GM-PET modality contains both structural MRI information and functional PET information. The details of our image fusion method include the following steps.

(a) Skull-stripping is performed on structural MRI scans using the “watershed” module in FreeSurfer 6.0 (29), as shown in Figure 2A. The watershed segmentation algorithm can strip skull and other outer non-brain tissue to produce the brain volume with much less noise and irrelevant information. As expected, the result, called SS-MRI, preserves only the intracranial tissue structure and removes areas of irrelevant anatomical organs.

(b) As shown in Figure 2B, SS-MRI is affine transformed to MNI152 space (30), a universal brain atlas template, using the FLIRT (FMRIB's Linear Image Registration Tool) module (31) in the FSL package. FLIRT is a fully automated robust and accurate tool for intra- and inter-modal brain image registration by linear affine (31, 32). The registration aims to remove any spatial discrepancies between subjects in the scanner and minimize translations and rotations from a standard orientation. This helps to improve the precision of the subsequent tissue segmentation. This registered MNI-MRI is used as the input modality to unimodal AD classification tasks.

(c) The GM area is segmented from the MRI scan using the FAST (FMRIB's Automated Segmentation Tool) module (33) in the FSL package. FAST segments a 3D brain image into different tissue types, while correcting for spatial intensity variations (also known as bias field or RF inhomogeneities). The underlying method is based on a hidden Markov random field model and an associated expectation-maximization algorithm. The whole automated process can produce a bias-field-corrected input image and probabilistic and/or partial volume tissue segmentation. It is robust and reliable compared with most finite mixture model-based methods, which are sensitive to noise. As shown in Figure 2C, the segmentation output of GM tissue is called GM-MRI.

(d) MNI-PET is obtained by co-registering the FDG-PET image to its respective MNI-MRI image using the FSL FLIRT module, as shown in Figure 2D. This gives the FDG-PET image the same spatial orientation, image size (for example, 182 × 218 × 182), and voxel dimensions (for example, 1.0 × 1.0 × 1.0 mm) as the MNI-MRI. After co-registration, the MNI-PET and MNI-MRI obtained are in the same sample space.

(e) The GM-MRI obtained in step (c) is used as an anatomical mask to cover the full MNI-PET image. MNI-GM-PET is obtained by a mapping operation, as illustrated in Figure 2E. So far, we have obtained the anatomical structure of GM on FDG-PET images. Nevertheless, compared with Origin-PET from coronal-axis and transverse-axis views, the mapped grayscale values in MNI-GM-PET images change significantly after MNI152 spatial registration; thus, they cannot reflect the true metabolic information as the Origin-PET does.

(f) In order to solve the grayscale deviation problem mentioned above, MNI-GM-PET is co-registered to the corresponding Origin-PET image, using the FSL FLIRT module, to obtain the GM-PET image, as shown in Figure 2F. On the one hand, this registration operation eliminates the deviation caused by affine transformation and preserves the true grayscale distribution of the original PET image; on the other hand, it ensures that the GM-PET has the same spatial size as the Origin-PET, that is, the MNI-GM-PET size of 182 × 218 × 182 is reduced to the original PET size of 160 × 160 × 96. This resolution reduction could also save computational time and memory costs.

### 2.3. Networks

At present, CNN is attracting increasing attention owing to its significant advantages in medical image classification tasks. In two-dimensional (2D) CNN approaches, where the 3D medical image is processed slice-by-slice, the anatomical context in directions orthogonal to the 2D plane is completely discarded. As discussed recently by (34), 3D CNN can greatly improve performance by considering the 3D data as a whole input, although the computational complexity and memory cost are increased owing to the larger number of parameters. To evaluate the effectiveness of the fused GM-PET modality in different CNNs, this paper introduces the 3D Simple CNN and 3D Multi-Scale CNN, designed by observing the characteristics of AD classification tasks, which will be explained in detail below.

#### 2.3.1. 3D Simple CNN

**Figure 3:**
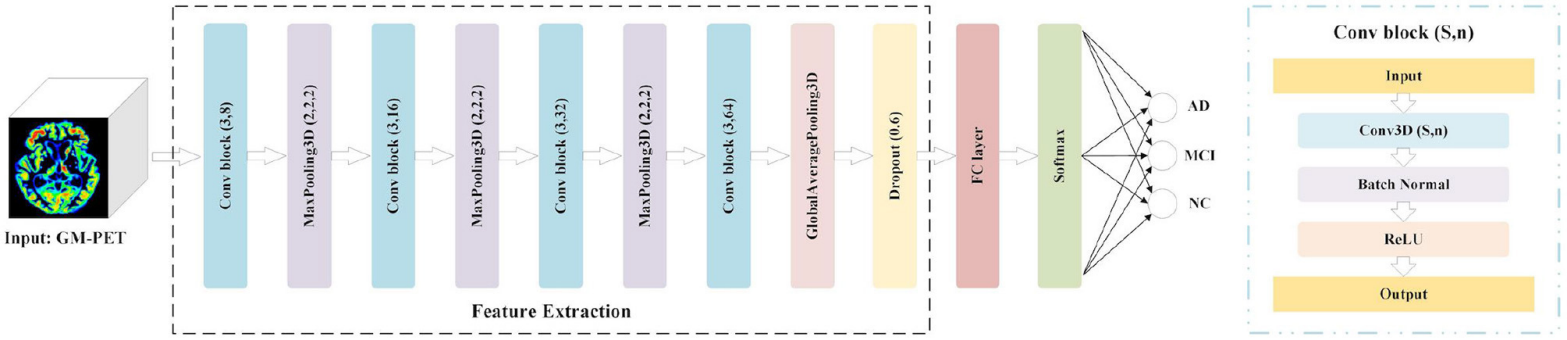
3D Simple CNN architecture for AD classification.

Considering the tradeoffs between the feature capture capabilities of 3D CNN and the potential overfitting risk caused by a small dataset, we propose a 3D Simple CNN to capture AD features from medical images. As shown in Figure 3, the 3D Simple CNN contains 11 layers, of which there are only four convolutional layers. Compared with deeper networks, the 3D Simple CNN has far fewer parameters and can better alleviate overfitting problems.

Specifically, the base building block, called Conv-block(*s, n*), consists of three serial operations: Conv3D(*s, n*), which stands for 3D convolution with *n* filters of *s* × *s* × *s* size, batch normalization (35), and a rectifier linear unit (ReLU). In this architecture, the “Feature Extraction” module is mainly composed of four Conv-blocks with parameters (3,8), (3,16), (3,32), and (3,64). That is, the convolution kernel sizes are (3, 3, 3), and the number of channels doubles in turn. There is also a 3D max-pooling layer with a pooling size of (2, 2, 2) between every two Conv-blocks. Besides, we add a global average pooling (GAP) layer and a dropout layer with a rate of 0.6 to avoid overfitting. After the Feature Extraction module, we connect an FC layer and a softmax layer for AD classification. In general, the 3D Simple CNN can be regarded as a baseline network for evaluating our image fusion method because of its plain structural composition.

#### 2.3.2. 3D Multi-Scale CNN

**Figure 4:**
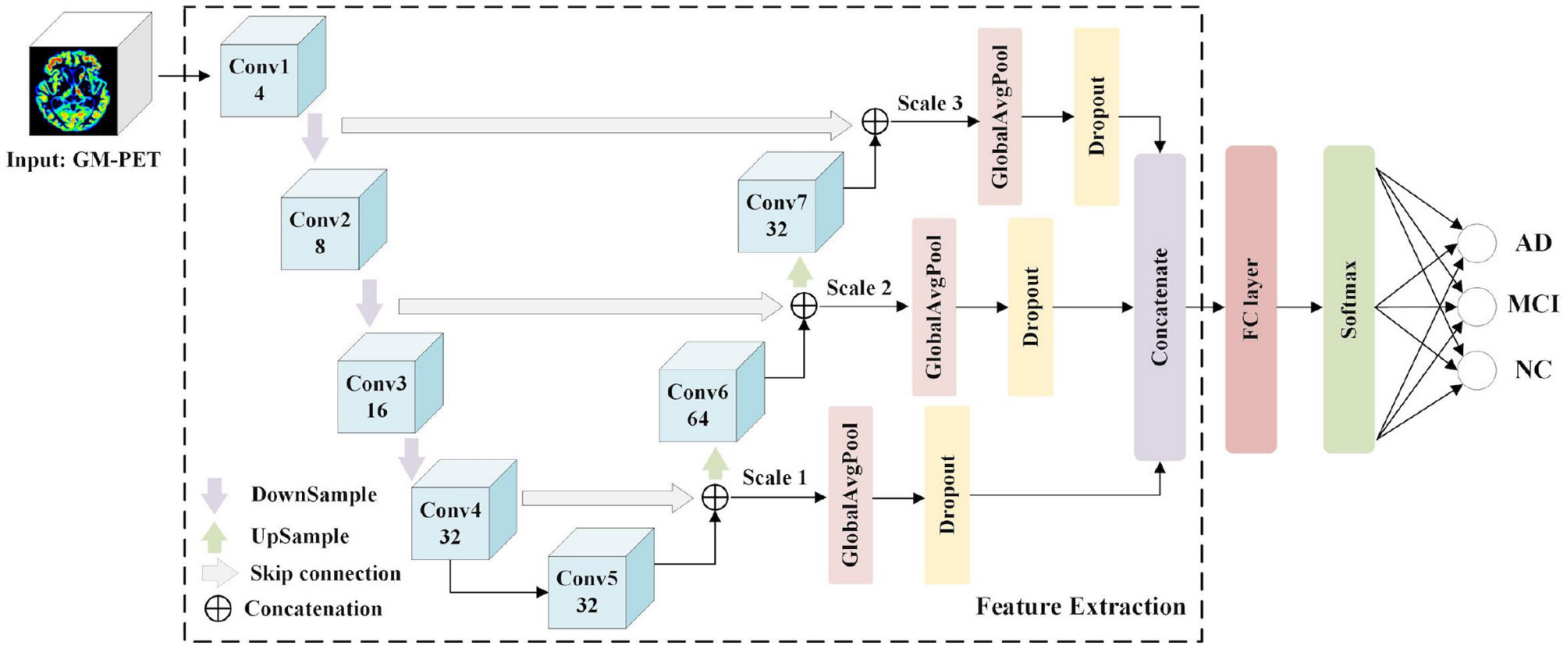
3D Multi-Scale CNN architecture for AD classification.

Numerous UNet-based networks have been proven effective in biomedical image recognition tasks (36–38), as the U-shaped network architecture with skip connections can obtain both relevant context information and precise location information. Motivated by the observation that features both from low-level image volumes and high-level semantic information can be obtained at different resolution scales, a 3D Multi-Scale CNN is proposed for AD classification, as shown in Figure 4.

The Feature Extraction module is used to extract and merge multi-scale features, and a classifier module consisting of an FC layer and a softmax layer predicts the group labels. The Feature Extraction module consists of seven convolutional layers (Conv1–Conv7) where the first four convolutional layers generate feature maps in a coarse-to-fine manner, and the last two layers (Conv6 and Conv7) are obtained by up-sampling the combined output of the “skip connection.” These convolutional layers are designed using a conventional CNN structure with kernel sizes of (3, 3, 3) and channel numbers as shown in Figure 4. Taking into account the overfitting problem, we properly reduce the channel numbers of convolutional layers. Detailed image features are often related to shallow layers, whereas semantically strong features are often associated with deep layers. It is desirable to obtain both types of features for AD classification by integrating information from different scales. Hence, the skip connection is used to combine features from both shallow and deep convolutional layers. More specifically, the down-sampled outputs of convolutional layers 1 and 2 are combined with the outputs of convolutional layers 7 and 6, respectively. Besides, the outputs of convolutional layers 4 and 5 are concatenated. Owing to the limitations of GPU memory when using 3D scans as inputs, three scales are used here. For each scale feature, we apply a GAP layer and a dropout layer to retain multi-resolution features, after which the outputs are concatenated to feed the following classifier. It is expected that multi-scale features with different levels of information will contribute to the diagnosis of AD.

## 3. Experiment and Results

### 3.1. Pre-processing

As inputs to CNN, 3D data with a generally high resolution would consume more computing resources during network training. Therefore, we process the input data using cropping and sampling operations to speed up the calculation of singleton data. (1) Cropping: As shown in Figure 2, there are many background areas with a pixel value of 0 outside the brain tissue area in each modality image. Without affecting the brain tissue regions, we appropriately reduce these meaningless background areas to decrease the size of the input data. Specifically, MRI is cropped from 182 × 218 × 182 to 176 × 208 × 176. In addition, PET and GM-PET are both cropped from 160 × 160 × 96 to 112 × 128 × 96. (2) Sampling: Each sample is divided into two by taking every other slice along the transverse axis. Concretely, the sizes of the MRI, PET, and GM-PET images become 176 × 208 × 88, 112 × 128 × 48, and 112 × 128 × 48, respectively. This can double the number of samples while reducing the resolution, which is conducive to better iteration and optimization of the network model.

### 3.2. Experimental Setup

In this paper, the networks involved are implemented in the Tensorflow (39) deep learning framework. We execute four classification tasks, i.e., AD vs. NC, AD vs. MCI, MCI vs. NC, and AD vs. MCI vs. NC, whereas previous studies such as (40) and (41) only classified AD vs. NC, which are the easiest groups to distinguish. We conduct comparative experiments on unimodal and multimodal data. For the network optimizer, Adam with an initial learning rate of 1e-4 is used to update the weights during training. The binary cross-entropy is applied as the loss function in the binary-classification task, whereas the categorical cross-entropy is used in the three-classification task.

We adopt a 10-fold cross-validation strategy to calculate the measures, so as to obtain a fairer performance comparison. We randomly divide the subjects in the dataset into 10 subsets, with one subset used as the test set, another subset used as the validation set, and the remaining eight subsets used as the training set. We train each experiment during 500 epochs and use two strategies to update the learning rate. (1) When the loss in the validation set does not decrease within 30 epochs, the learning rate drops to one-tenth of the current level. (2) When the accuracy in the validation set does not increase within 20 epochs, the learning rate is reduced by half. At the same time, an early stopping strategy is applied. That is, the training is stopped if the loss on validation does not decrease within 50 epochs. The classification accuracy (ACC), sensitivity (SEN), and specificity (SPE) are selected as the evaluation measures. We report the results as the mean ± SD (standard deviation) of the 10-fold tests.

**Figure 5:**
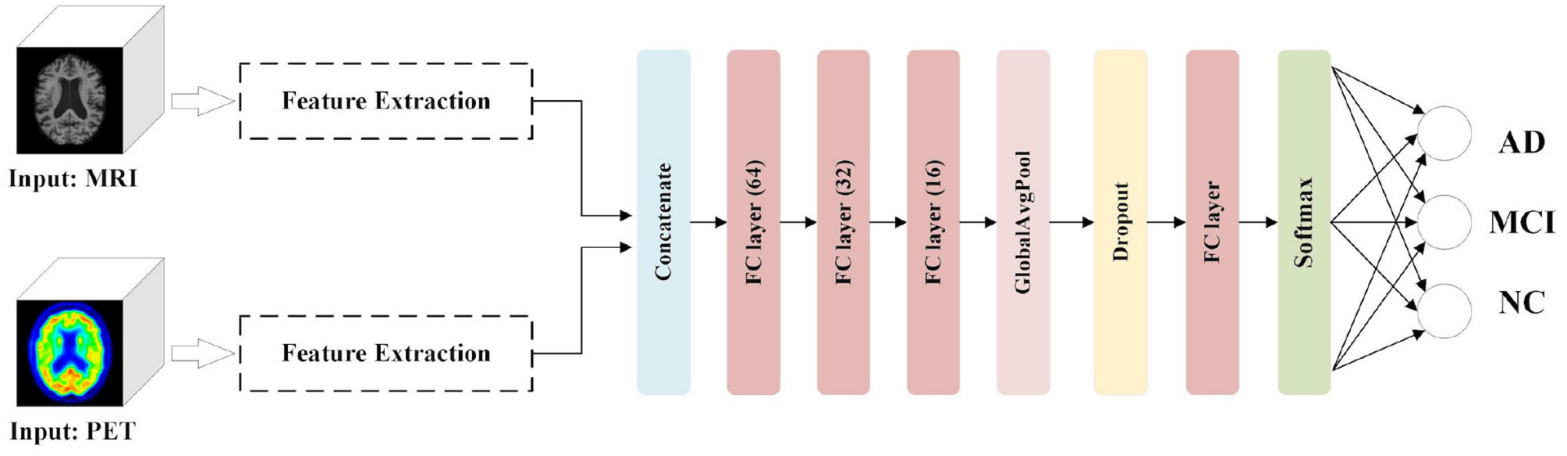
AD diagnostic framework with multimodal feature fusion method.

We aim to comprehensively evaluate the effectiveness of our image fusion method in the proposed diagnostic framework for AD classification tasks. In addition to considering other unimodal scans (for example, MRI and PET) as inputs, we present an AD diagnostic framework with the feature fusion method as a benchmark. As shown in Figure 5, the Feature Extraction module is used to obtain semantic information from the 3D volumes of MRI and PET images, respectively. After the extracted features are concatenated, three FC layers with unit numbers of 64, 32, and 16, respectively, perform the correlation fusion. Moreover, a GAP layer and a dropout layer are applied to avoid overfitting. Finally, the classification module, which consists of an FC layer and a softmax layer, predict the group labels.

### 3.3. Performance

#### 3.3.1. Results for AD vs. NC

In the classification of AD vs. NC, Table 2 shows the results of unimodal and multimodal modalities with different networks. The multi-modality-based methods such as the feature fusion method and the proposed image fusion method achieve better performance, because they successfully fuse MRI and PET information. Between the two multimodal methods, our image fusion method has better overall indicators. With the 3D Simple CNN, our image fusion method obtained the best classification accuracy of 94.11 ± 6.0% and specificity of 95.04 ± 5.7%, and the second best sensitivity of 92.22 ± 6.7%. The feature fusion method achieved the best sensitivity of 94.44 ± 7.9% but showed lower accuracy and specificity. With the 3D Multi-Scale CNN, the proposed image fusion method for AD diagnosis achieved the best classification accuracy of 94.11 ± 4.0%, sensitivity of 93.33 ± 7.8%, and specificity of 94.27 ± 6.3%. Moreover, it showed improvements in classification accuracy, sensitivity, and specificity over the unimodal methods of at least 4.75, 6.27, and 3.46%, respectively. Overall, our image fusion method achieved the overall best performance in the AD vs. NC classification task.

**Table 2:** Results of different modalities with different networks for AD vs. NC (UNIT:%).

**Network**	**Modalities**	**ACC**	**SEN**	**SPE**
3D Simple CNN	Unimodal MRI	89.80 ± 4.7	86.31 ± 12.0	91.97 ± 5.5
	Unimodal PET	92.10 ± 5.8	89.13 ± 9.7	94.27 ± 4.1
	Feature fusion	93.22 ± 3.8	**94.44** **±** **7.9**	91.62 ± 7.5
	Proposed image fusion	**94.11** **±** **6.0**	92.22 ± 6.7	**95.04** **±** **5.7**
3D Multi-Scale CNN	Unimodal MRI	88.88 ± 6.8	86.11 ± 13.9	90.43 ± 4.5
	Unimodal PET	89.36 ± 9.1	87.06 ± 16.3	90.81 ± 7.5
	Feature fusion	93.66 ± 5.3	93.33 ± 9.4	93.50 ± 6.3
	Proposed image fusion	**94.11** **±** **4.0**	**93.33** **±** **7.8**	**94.27** **±** **6.3**

*Bold value mean the best indicator value under the same conditions*.

#### 3.3.2. Results for MCI vs. NC

Table 3 shows the results for different modalities in the classification of MCI vs. NC with different networks. The proposed image fusion method showed significant performance superiority. With the 3D Simple CNN, our image fusion method achieved the best classification accuracy of 88.48 ± 6.5%, sensitivity of 93.44 ± 6.5%, and specificity of 82.18 ± 12.3%. It also showed improvements in classification accuracy, sensitivity, and specificity over the feature fusion method of at least 6.11, 1.25, and 11.62%, respectively, indicating that the proposed image fusion method fuses multimodal information in a more effective way. When applying the 3D Multi-Scale CNN, our image fusion method still achieved the best accuracy of 85.00 ± 9.4% and specificity of 85.60 ± 11.7%, and the second best sensitivity of 84.69 ± 12.5%. In terms of specificity, our method far exceeded other methods by at least 11.33%. Generally speaking, the proposed image fusion method achieved the overall best performance in the MCI vs. NC classification task.

**Table 3:** Results of different modalities with different networks for MCI vs. NC (UNIT:%).

**Network**	**Modalities**	**ACC**	**SEN**	**SPE**
3D Simple CNN	Unimodal MRI	79.46 ± 9.4	87.50 ± 16.1	69.15 ± 10.7
	Unimodal PET	72.00 ± 7.8	72.81 ± 10.5	70.56 ± 12.2
	Feature fusion	82.37 ± 9.0	92.19 ± 13.1	69.74 ± 18.0
	Proposed image fusion	**88.48** **±** **6.5**	**93.44** **±** **6.5**	**82.18** **±** **12.3**
3D Multi-Scale CNN	Unimodal MRI	76.01 ± 8.8	77.50 ± 13.4	74.27 ± 9.7
	Unimodal PET	68.55 ± 5.4	65.94 ± 13.5	70.64 ± 14.8
	Feature fusion	83.17 ± 6.5	**90.63** **±** **15.7**	73.55 ± 16.7
	Proposed image fusion	**85.00** **±** **9.4**	84.69 ± 12.5	**85.60** **±** **11.7**

*Bold value mean the best indicator value under the same conditions*.

#### 3.3.3. Results for AD vs. MCI

In the classification of AD vs. MCI, Table 4 shows the results of unimodal and multimodal modalities with different networks. With the 3D Simple CNN, our image fusion method for AD diagnosis achieved the best classification accuracy of 84.83 ± 7.8% and specificity of 94.69 ± 6.3%, and the second best sensitivity of 68.29 ± 19.8%. Moreover, the proposed image fusion method showed improvements in classification accuracy, sensitivity, and specificity over the unimodal methods by at least 6.53, 10.83, and 5.00%, respectively. With the 3D Multi-Scale CNN, our image fusion method obtained the best classification accuracy of 80.80 ± 5.9% and sensitivity of 71.19 ± 14.6%, and the second best specificity of 85.94 ± 11.8%. Compared with the feature fusion method, which achieved the best specificity, the proposed image fusion method showed improvements in classification accuracy and sensitivity of 0.33 and 17.78%, respectively. On the whole, our method outperformed the other methods and showed the best overall performance in the AD vs. MCI classification task.

**Table 4:** Results of different modalities with different networks for AD vs. MCI (UNIT:%).

**Network**	**Modalities**	**ACC**	**SEN**	**SPE**
3D Simple CNN	Unimodal MRI	72.47 ± 7.8	46.59 ± 18.8	87.50 ± 12.1
	Unimodal PET	78.30 ± 10.3	57.46 ± 20.1	89.69 ± 10.9
	Feature fusion	81.00 ± 8.1	**68.33** **±** **15.3**	88.75 ± 9.2
	Proposed image fusion	**84.83** **±** **7.8**	68.29 ± 19.8	**94.69** **±** **6.3**
3D Multi-Scale CNN	Unimodal MRI	68.40 ± 8.4	52.70 ± 19.7	77.50 ± 11.9
	Unimodal PET	73.07 ± 15.3	61.90 ± 27.6	79.38 ± 16.9
	Feature fusion	80.47 ± 9.4	53.41 ± 25.1	**95.94** **±** **5.1**
	Proposed image fusion	**80.80** **±** **5.9**	**71.19** **±** **14.6**	85.94 ± 11.8

*Bold value mean the best indicator value under the same conditions*.

#### 3.3.4. Results for AD vs. MCI vs. NC

Table 5 shows the results of different modalities for the classification of AD vs. MCI vs. NC with the 3D Simple CNN and 3D Multi-Scale CNN. As MCI is a transitional state between AD and NC, many confounding factors are introduced in the multi-class task. Clearly, the classification task of AD vs. MCI vs. NC is more difficult than the above binary-classification tasks. In this case, our image fusion method still showed the best performance on all evaluation indices, whereas the unimodal and feature fusion methods were particularly lacking in power for the three-classification task. With the 3D Simple CNN, the best classification accuracy, sensitivity, and specificity were 74.54 ± 6.4, 59.41 ± 8.2, and 85.41 ± 4.2%, respectively. Compared with other methods, our image fusion method showed improvements in classification accuracy, sensitivity, and specificity of at least 9.06, 10.73, and 6.27%, respectively. With the 3D Multi-Scale CNN, our image fusion method achieved the best classification accuracy of 71.52 ± 5.0%, sensitivity of 55.67 ± 6.2%, and specificity of 83.40 ± 3.3%. Furthermore, our image fusion method showed improvements in classification accuracy, sensitivity, and specificity over the other methods of at least 3.37, 4.03, and 2.37%, respectively. Clearly, our image fusion method showed significant advantages in the multi-class task.

**Table 5:** Results of different modalities with different networks for AD vs. MCI vs. NC (UNIT:%).

**Network**	**Modalities**	**ACC**	**SEN**	**SPE**
3D Simple CNN	Unimodal MRI	64.00 ± 8.6	47.10 ± 9.5	78.08 ± 6.5
	Unimodal PET	60.65 ± 9.7	43.50 ± 10.6	75.49 ± 7.3
	Feature fusion	65.48 ± 5.9	48.68 ± 6.7	79.14 ± 4.3
	Proposed image fusion	**74.54** **±** **6.4**	**59.41** **±** **8.2**	**85.41** **±** **4.2**
3D Multi-Scale CNN	Unimodal MRI	66.24 ± 5.9	49.56 ± 6.6	79.72 ± 4.3
	Unimodal PET	59.98 ± 7.1	42.83 ± 7.0	74.98 ± 5.9
	Feature fusion	68.15 ± 9.4	51.64 ± 10.5	81.03 ± 6.9
	Proposed image fusion	**71.52** **±** **5.0**	**55.67** **±** **6.2**	**83.40** **±** **3.3**

*Bold value mean the best indicator value under the same conditions*.

#### 3.3.5. Comparisons With State-of-the-Art Methods

The proposed image fusion method was evaluated and compared with the state-of-the-art multimodal approaches for each task-specific classification (Table 6). The results indicate that our method (Image Fusion + 3D Simple CNN) achieved the highest accuracy and outperformed other multimodal methods for each AD diagnostic task. Although our multimodal image fusion method is time-consuming during the pre-processing steps, the network parameters are greatly reduced because only the composite image is fed into the classification network instead of a set of images of different modalities. In other words, the computation complexity and the memory cost of the proposed image fusion method are no higher than those of competing methods.

**Table 6:** Comparative performance of our classifiers vs. competitors. Numbers in parentheses denote the numbers of AD/MCI/NC subjects in the dataset used.

**Approach**	**Dataset**	**Accuracy (%)**			
		**AD vs. NC**	**MCI vs. NC**	**AD vs. MCI**	**AD vs. MCI vs. NC**
(42)	MRI+PET (85/169/77)	91.4	82.1	–	53.79
(20)	MRI+PET (51/99/52)	91.4	77.4	70.1	–
(21)	MRI+PET+CSF+Genetic (37/75/35)	91.8	79.5	–	60.2
(23)	MRI+PET (238/217/360)	84.59	85.96	–	–
(24)	MRI+PET (93/204/100)	93.26	74.34	–	–
(10)	MRI+PET+CSF (210/541/160)	88.02	84.14	–	–
(43)	MRI+PET (160/187/160)	92.51	82.53	–	–
(19)	fMRI+SNP (37/37/35)	81.0	80.0	–	–
Our Method (Image Fusion+3D Simple CNN)	MRI+PET (95/160/126)	**94.11**	**88.48**	**84.83**	**74.54**

*Bold value mean the best indicator value under the same conditions*.

### 3.4. Visualization

**Figure 6:**
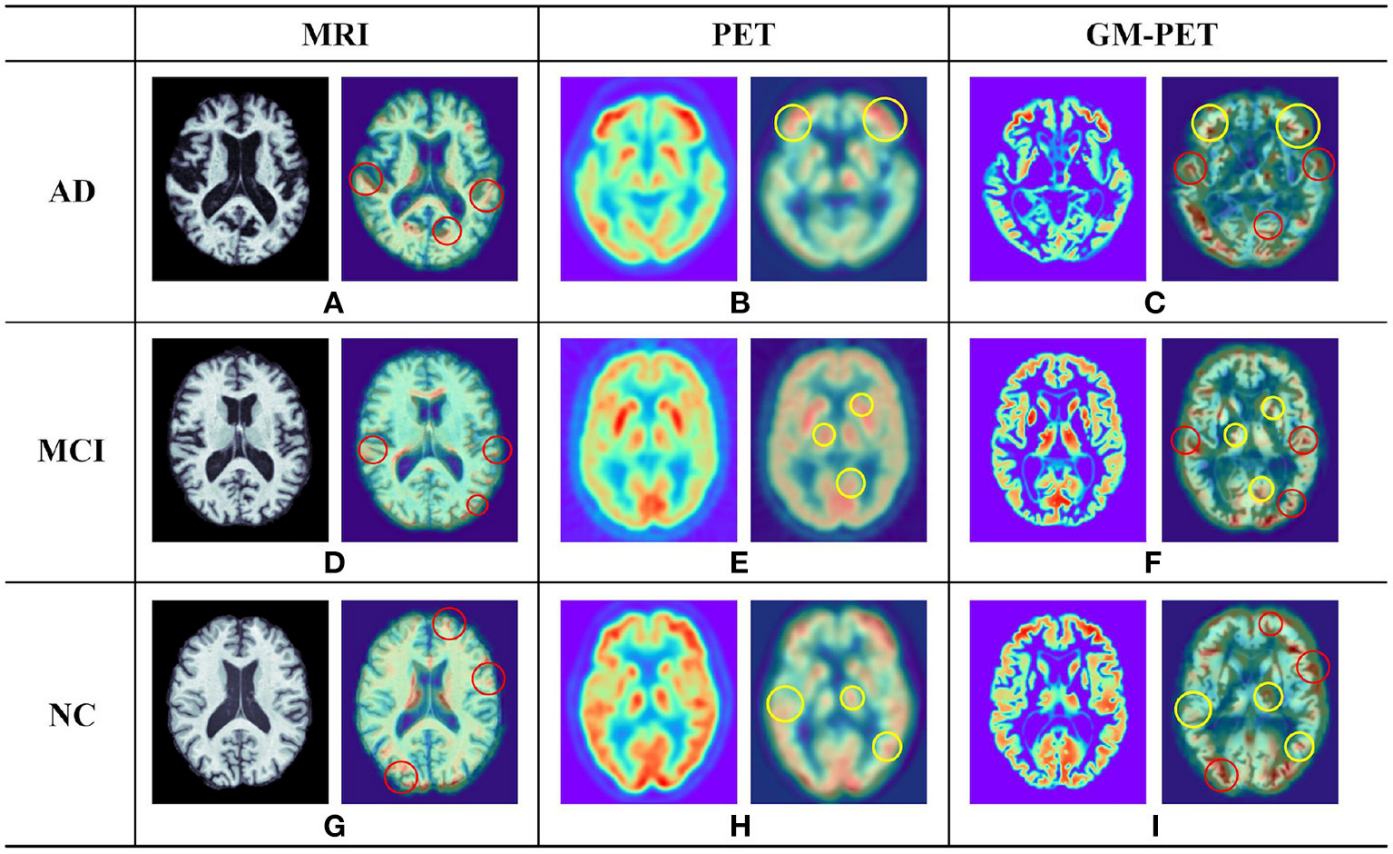
Examples of different modality images for AD, MCI, and NC subjects. In each of the nine cells **(A–I)**, the picture on the left is a subject slice and the picture on the right is the Grad-CAM result for that slice. The red circle in the 3D Grad-CAM results outlines the contour areas of common interest in the MRI and GM-PET images, while the yellow circle outlines the metabolic characteristic areas of common interest in the PET and GM-PET images.

To further illustrate the plausibility of our image fusion method, we visualized origin images and the corresponding features in different modalities for different subject groups, as shown in Figure 6. The picture on the left in each cell is a slice of the subject in different modalities. From the MRI and PET modality slices, we observed that the AD subject had the most obvious brain tissue loss and decrease in metabolism, respectively, followed by the MCI subject, whereas the NC subject had a healthy brain imaging scan. From the GM-PET slices, we observed that the GM area was delineated while maintaining the same pattern as that of the PET modality. GM-PET well-inherited the ability of MRI to express atrophy of brain tissue and the ability of PET to observe metabolic levels. As only the GM region was retained, there was no noise information around the brain tissue in the GM-PET images; in particular, the irrelevant skull area was cleanly removed. Based on the richness of the information expressed by the images, there is no doubt that our proposed image fusion method achieved better results.

It was worth investigating whether the multimodal GM-PET provided the feature extraction module of the CNN with ample information. We applied 3D Grad-CAM technology (44) to visualize the region of interest in the second convolutional layer of the 3D Simple CNN, shown as the right picture of each cell in Figure 6. The highlighted areas in the output images of Grad-CAM represent the key areas on which the convolutional layer focuses. In the outputs of the MRI slices, the focus was on the contour and edge texture areas, as outlined by the red circles. In the outputs of the PET slices, the areas of interest were highly consistent with the areas of high metabolic levels, as represented by the yellow circles. As expected, the convolutional layer on GM-PET considered both contour and metabolic information at the same time. Namely, the GM-PET modality provides more abundant characteristics for AD diagnosis.

## 4. Discussion

As multimodal data can provide more comprehensive pathological information, we propose an image fusion method to effectively merge the multimodal neuroimaging information from MRI and PET scans for AD diagnosis. Based on the observation that GM is the tissue area of most interest in AD diagnostic researches (10, 11, 45), the proposed fusion method extracts and fuses the GM tissue of brain MRI and FDG-PET in the image field so as to obtain a fused GM-PET modality. As can be seen from the image fusion flow, shown in Figure 2, the GM-PET image not only reserves the subject's brain structure information from MRI but also retains the corresponding metabolic information from PET. With the 3D Grad-CAM technology, we observe that the convolutional layer that extracts the GM-PET features can capture both contour and metabolic information, indicating that the GM-PET modality can indeed provide richer modality information for classification tasks. Moreover, our proposed image fusion method, through its registration operation, better solves the heterogeneous features alignment problem between multimodal images, compared with methods based on multimodal feature learning.

In addition, the 3D Simple CNN and 3D Multi-Scale CNN are presented to perform four AD classification tasks, comprising three binary-classification tasks, i.e., AD vs. NC, AD vs. MCI and MCI vs. NC, and one multi-classification task, AD vs. MCI vs. NC. The 3D Simple CNN, with a plain structure, was proposed first as a baseline network. Then we proposed a 3D Multi-Scale CNN network that combines information from different scale features while capturing context information and location information. In order to prevent over-fitting, we designed these two networks using the following strategies: 1) Use fewer convolutional layers; (2) reduce the number of channels of the convolutional layer; (3) use GAP and dropout layers to reduce redundant information. Furthermore, the proposed AD diagnostic framework uses a single-input network instead of the multiple-input network used in feature fusion methods, as our image fusion method fuses multimodal image scans into a single composite image. Therefore, our image fusion method can greatly reduce the number of CNN parameters.

Extensive experiments and analyses were carried out to evaluate the performance of our proposed image fusion method. According to the classification results shown in Tables 2–5, the multimodal methods, including feature fusion and the proposed image fusion method, achieved better performance than the unimodal methods, as the multimodal methods contained abundant and complementary information. Our image fusion method outperformed the feature fusion method, especially in the complex three-classification task. Moreover, both the 3D Simple CNN and 3D Multi-Scale CNN produced consistent results indicating that our image fusion method had the best overall performance, with great adaptability to different classification networks. And our image fusion method also achieved better performance compared with the state-of-the-art multimodal-learning-based methods. Although the proposed image fusion method always showed the best accuracy, sometimes its performance was not optimal in terms of sensitivity and specificity. In order to solve this problem, we will further focus on WM and CSF tissues and combine their information with the existing GM information to provide better support for AD auxiliary diagnosis in the future.

## 5. Conclusion

We propose an image fusion method to combine MRI and PET scans into a composite GM-PET modality for AD diagnosis. The GM-PET modality contains both brain anatomic and metabolic information and eliminates image noise subtly so that the observer can easily focus on the key characteristics. To further evaluate the applicability of the proposed image fusion method, 3D Grad-CAM technology was used to visualize the area of interest of the CNN in each modality, showing that both the structural and functional characteristics of brain scans were included in the GM-PET modality. A series of evaluations based on the 3D Simple CNN and 3D Multi-Scale CNN confirmed the superiority of the proposed image fusion method. In terms of experimental performance, our proposed image fusion method not only overwhelmingly surpassed the unimodal methods but also outperformed the feature fusion method. Besides, the image fusion method showed better performance than other competing multimodal learning methods described in the literature. Therefore, our image fusion method is an intuitive and effective approach for fusing multimodal information in AD classification tasks.

## Data Availability Statement

The original contributions presented in the study are included in the article/supplementary material, further inquiries can be directed to the corresponding author/s.

## Author Contributions

JS wrote the main part of the manuscript. JZ proposed the key image fusion approach. PL and XL carried out the experiments and analyzed the results. GZ and PS built the AD diagnostic framework based on 3D CNN. All authors read and approved the final manuscript.

## Conflict of Interest

The authors declare that the research was conducted in the absence of any commercial or financial relationships that could be construed as a potential conflict of interest.
